# STATE-LEVEL EDUCATION QUALITY AND DEMENTIA INCIDENCE IN THE UNITED STATES

**DOI:** 10.1093/geroni/igae098.2364

**Published:** 2024-12-31

**Authors:** Hyungmin Cha, Mateo Farina, Heide Jackson, Jennifer Ailshire, Katrina Walsemann

**Affiliations:** University of Southern California, Los Angeles, California, United States; University of Texas at Austin, Austin, Texas, United States; University of Maryland, College Park, Maryland, United States; University of Southern California, Los Angeles, California, United States; University of Maryland, College Park, Maryland, United States

## Abstract

Despite the robust link between education and dementia risk, less is known about how state education quality relates to dementia, particularly regarding how it differs by race. Drawing on data from the Health and Retirement Study (2000-2018) of adults aged 55 years and older, linked to historical state school investment records from 1914 to 1959, we employed hazard models to examine the association between state-level education quality and dementia onset. Our findings reveal a significant association between state education quality and dementia risk, and this association remains consistent across both White and Black adults. These results suggest that state investment in public education may have long-ranging influences on dementia onset, with consistent effects observed across racial subpopulations.

